# Correspondence

**Published:** 1866-07

**Authors:** James P. White

**Affiliations:** Rome


### Correspondence.

Rome, May 21st, 1866.

To the Editor of the Buffalo Medical and Surgical Journal:

My Dear Doctor:—From Granada in Spain, whence I last wrote you, we coasted the Mediterranean, by rail, by diligence or by vettura to Genoa in Italy. This route passes through countries surpassingly beautiful and interesting. In the course of its progress it affords almost every conceivable variety of scenery. In this journey, which is more than one thousand miles in length by this route, which is ordinarily pursued by the tourist, we passed for the most part in close proximity to the sea on the one hand, whilst upon the other, we had a succession of artificial terraces extending high up into the mountains, the summits being surmounted by peaks of granite. These terraces, stolen as it were from their barren hill-side, are ever covered with the vine, the orange, the lemon, the fig, and still more abundantly with the olive. The road is often carried along upon high cliffs overhanging the sea and commanding beautiful views of these historic waters.

Between Nice and Genoa we passed over the famous "*Cornici*" road, which now occupies the site, during most of its route, of the older "Aurelian way." The present road was built by the French and Sardinian governments, and is a most permanent and beautiful structure. It is carried along the slopes of the sub-Alpine chain, as it forms the shores; in some parts rising to a great height, and although doubtless entirely safe, it hangs upon the margin of the precipice so as to excite the apprehensions of the nervous traveler. Passing over this road in the latter part of the month of March, whirled along at a rapid rate, in a vettura, with a pleasant party of four, and drawn by four fine horses, through picturesque scenery, and stopping at discretion at good hotels, in quaint old castles or villas, with uninterrupted sunshine, constitutes, in my experience, the "poetry of traveling." The towns and villages, thickly studded along the coast, and glittering upon the sides of the hills, sometimes placed at a great elevation, wear a gay aspect. The old churches, which are to be found in every cluster of houses, no matter how small the village, have usually very lofty facades, painted in fresco. At a distance, the lofty and bold elevations, the gay colors, the tall belfreys, and numerous cupolas, produce a striking effect upon the beholder, who only detects any defects arising from profusion of gay ornamentation upon a near approach. Suddenly you rush into one of these old towns with streets so narrow as not to permit a wheelbarrow to pass you, driving the foot passengers rapidly before you or compelling them to take shelter in the shops on either side. This coast or "*riverid*," as it is called, sloping toward the sea, and exposed to a southern sun, enjoys a temperature scarcely to be surpassed. Nice, Mentone, San Remo and Bordighiera, have a world-wide reputation as winter residences for patients with pulmonary difficulties.

But I must not linger in the orange groves or among the beautiful cypresses of this enchanting coast; nor must I ask the musical reader to accompany me to dreamy Venice, "Queen of the Sea," and float among her princely palaces to the song of the gondolier, feasting his eyes upon the treasures of art which they contain, as he glides through its liquid streets. No, the true disciple of Esculapius is compelled in the practice of his arduous profession to forego all indulgence of his poetic fancy and confine himself to sober, scientific realities. He has sworn fidelity in a profession which recognizes no night, no Sunday, no season of rest or holiday, which heeds not the storm, and seeks the pestilence with all its terrors; he can scarcely afford time to accompany an idle brother into these fairy scenes. Omitting then all allusion to beautiful statuary and paintings, to splendid palaces and magnificent churches, to be found in all the large cities of Italy, let me ask him to accompany me through the halls of the old Universities of Padua and Bologna.

The University, or in more ancient language, the *s ud o* of Padua, enjoyed considerable celebrity more than six hundred years ago. At first it was prominent in law, and the great Baldus here taught and professed what lawyers call "the written reason." Padua also greatly excelled in medicine, and the medical professorships of the University include some of the greatest names of the sixteenth and seventeenth centuries. Tesalius Fallopius, Fabricius de Aquapendente, Spigelius and Sanctorius here taught with great distinction in the sixteenth and early part of the seventeenth centuries. In times much nearer to our own, Morgagni continued to emulate their honors, making anatomical discoveries which will hand his name down to posterity. This University was especially encouraged and protected by the Venetians, and with the decline of the city and republic of Venice, this school lost its great patron, and soon after its exalted position, as a seat of learning. Padua can boast the earliest anatomical theatre, and the most ancient botanic garden in existence. The latter was instituted by the Venetian Senate as early as 1543. The collection of anatomical models in the museum is still worthy attention, and the garden is interesting as containing some of the oldest specimens of trees and plants now common in Europe—patriarchs of other gardens and conservatories. These magnificent old halls, however, are now nearly deserted, and the crowds who formerly listened to Spigelius and Morgagni have forsaken these ancient and spacious apartments, for Paris, Vienna and London.

We next proceeded, by the way of Ferrara, to the old city of Bologna, in former times the second capital of the States of the Church, and the seat of an Archbishopric. The University of Bologna, celebrated as the oldest in Italy, and as the first in which academical degrees were conferred, was long the glory of its citizens. It was founded by Irnercus in 1119, who taught the law with such reputation in his native city, that he acquired the title of "Lucirna Juris." During the troubled period of the twelfth century the fame of this University attracted students frcm all parts of Europe. I was assured, by the old custode of the University, that for several consecutive years in the thirteenth century the number of students exceeded 18,000, and that at no time for more than one hundred years did it fall below 10,000. In the fourteenth century it acquired lasting celebrity as the first school which practiced dissection of the human body, without which anatomy can be but very imperfectly taught. In more recent times it became renowned for the discovery of galvanism within its walls; and to its credit it may be added, a statue has been erected to the memory of its discoverer. The University buildings, lecture rooms and library are the most spacious and best arranged in many respects of any which I have ever seen. The library rooms are far more spacious and better lighted than the Bodleau library of Oxford, England, or the Biblioteke Imperial in Paris, and contains 140,000 volumes, and 9,000 manuscripts.

This University has been remarkable for an *honor* peculiarly its own—the large number of its learned female professors. In the fourteenth century, Novella d'Andrea, daughter of the celebrated canonist, frequently occupied her father's chair, and it is recorded by Christiana de Piou, that her beauty was so striking that a curtain was drawn before her in order not to distract the attention of the students. Moore says:

> "Drawn before her,
> Lest, if her charms were seen, the students
> Should their young eyes wander o'er her,
> And quite forget their jurisprudence."

The modern specimens of strong minded women scarcely require this precaution. The name of Laura Bossi, professor of mathematics, is of more recent date. Still more astonishing to Americans is the instance of Monzolina, who graduated in surgery, and subsequently distinguished herself as professor of anatomy. Her statue is preserved, and may be seen among many of her learned *brethren*, occupying a niche in the Anatomical theatre.

This University, like that of Cordova in Spain, and that in Padua in the north of Italy, has lost its high reputation. Medical studies appear now to have the superiority which is in a great measure attributable to clinical teaching. Tommassins was among the first to teach at the bed-side and deservedly gave a high reputation to its clinical school which has been well maintained by his successors. The city is large enough and the authority liberal enough to afford the students ingress into hospitals of considerable extent for that purpose.

From Bologna we proceeded to Florence where we examined with care the great collection of wax preparations in the museum originally placed there by the Grand Duke. This is perhaps the most extensive as well as one of the most accurate and varied to be found in Europe. There are two or three hospitals, but so far as I could judge indifferently managed, and offering little inducement for the detention of the medical observer. The Italians are a non-progressive people, and this seems no less true in medical matters than in all the affairs of life, and you find yourself constantly reminded of the tardy advancement referred to in Spain in a letter from Cadiz.

Rome next claims a few remarks, not because her medical resources bear any proportion to her inexhaustable treasures in art and in ancient and modern architecture. Indeed Rome forms no exception to the other Italian cities in the present status of the medical profession. Upon visiting her largest hospitals, San Spirits, St. James, St. John, Lotteron, and the military hospitals, they were found badly ventilated and dirty. Little attention was paid to the convalescents in respect to their personal habits, and they seemed filthy and the odor of the wards exceedingly offensive. I was taken by Dr. Fidele, a medical practitioner of distinction in Rome, who has passed much time in London, and speaks the English language fluently, to see the hospital of the "Brothers of

Good Deeds," which forms a most remarkable exception to those already referred to. This institution is founded as a receptacle for the invalid of the better classes, and is a model of cleanliness, ventilation and good order. It is under the direction of the brotherhood who take great pride in the management of their charge. Such lofty ceilings, highly polished floors, luxurious bathing arrangements, elaborate kitchen furniture, and such an extensive laboratory I have never seen in a hospital elsewhere, and should it be my lot to fall ill in the "Eternal City," I should beg to be taken to these spacious apartments and committed to the good offices of the "brothers of good deeds."* They have a large garden in a high state of cultivation, with fountains constantly playing, attached to the institutions in which convalescents may exercise at discretion. It should be added that upon examination of their official reports, I find the per centage of mortality varying in different years from 3½ to only a little more than 8 per cent. This institution forms an honorable and most conspicuous exception to most Italian hospitals. The diseases most prevalent in Rome are miasmatic in their origin and intermittent in character. All are doubtless aware that in the immediate vicinity of this city, thousands of acres are now left uncultivated which once supported a dense population. These plains or *campagnia* are exceedingly fertile, producing in great abundance the most valuable products of this fruitful country. With slight exceptions the "Pontine marshes" only, the campagna is not covered with water, and even these might, without great difficulty, be drained into the sea. So might, in my opinion, by a little Yankee enterprise, this whole country be reclaimed and made immensely valuable, greatly improving the health of the city thereby. It requires to be treated as the prairies of Illinois have been, and the result upon the health of the inhabitants would be analagous. Plough it deeply so as to expose the vegetable mould to the air, drain the marshes, and above all plant trees so as to afford shade, and which by the absorbing surface afforded by their foliage and the wall of inter-

* It ought to be stated for the benefit of all Americans who may chance to require medical services in Rome, that Dr. James B. Gould, who served fifteen years as surgeon in the U. S. Navy, now resides there. Dr. G. is a highly intelligent and courteous gentleman, to whom I am indebted for many kindnesses, and whom I can unhesitatingly commend to the confidence of all my countrymen.

ruption which they supply, neutralize and arrest the malarious exhalations. During a few years and until the soil should be, as it were, regenerated and the forests somewhat grown, it would be well doubtless in reference to the hygiene of the pioneer to build elevated sleeping rooms, and require him to avoid twilight exposure. There can be little question that with proper sanitary measures and precautions the effort to reclaim these immense trackless, houseless wastes would be successful, and if situated near some large American city would be undertaken without hesitation.—They were deserted by the population for more secure mountain homes, at a time when the country was overrun by bandits and barons, scarcely less ferocious. Almost all the walled towns and strong holds of Italy are situated upon the summits of hills or rocks which are approached only with difficulty, and frequently by zigzags cut in the solid rock. Into these defensible, castellated towns, all the inhabitants of the surrounding campagnia were driven during the middle ages by the feudal wars between the different barons and by the roving bands of marauding robbers. Meanwhile the uninhabited low lands became a waste, and the constant decomposition of the annual growth of vegetable matter gave rise to those noxious exhalations which produce the fevers of that country. Re-opening the drains with suitable sub-soil drainage and cultivation of the soil, and the growth of forest trees would then, in my opinion, render the campagnia habitable and greatly improve the healthfulness of Rome which it surrounds.

But, Mr. Editor, I find myself inclined to indulge in a prosaic essay upon the sanitary condition of this ancient country, which would possess little interest to most of your readers, and will therefore spare them the infliction by abruptly subscribing myself,

Truly yours, JAMES P. WHITE.

